# Folliculosebaceous Cystic Hamartoma of the Eyelid: A Case Report

**DOI:** 10.7759/cureus.89853

**Published:** 2025-08-12

**Authors:** Akruti S Desai, Chitra Madiwale

**Affiliations:** 1 Department of Ophthalmic Plastic Surgery, Eye Cancer &amp; Aesthetic Services, Shantilal Shanghvi Eye Institute, Mumbai, IND; 2 Department of Lab Medicine, P.D. Hinduja Hospital and Medical Research Centre, Mumbai, IND

**Keywords:** eyelid mass, eyelid tumours, folliculosebaceous cystic hamartoma, sebaceous gland carcinoma, sebaceous trichofolliculoma

## Abstract

Folliculosebaceous cystic hamartoma (FSCH) is a cystic cutaneous entity characterized by a cystic folliculosebaceous development accompanied by mesenchymal components, often comprising adipocytes, variable fibrous stroma, and small vascular channels. We report a case of a 66-year-old female patient with a painless lesion on her right upper eyelid causing mechanical ptosis. On the tarsal surface of the eyelid, a fungating mass with a prominent vessel and yellowish deposits was visible. The differential diagnosis considered was that of a benign sebaceous eyelid mass, such as a sebaceous hamartoma or trichofolliculoma, and sebaceous gland carcinoma. The patient underwent complete excision of the lesion with eyelid reconstruction.

Histopathologically, it exhibited a well-circumscribed, unencapsulated lesion with a central dilated cystic space lined by attenuated squamous epithelium, containing sparsely distributed keratin flakes, and surrounded by numerous sebaceous lobules. The background fibrous stroma showed numerous foamy histiocytes, smaller empty dilated cystic spaces, spindle cells, and fat spaces. FSCH of the eyelid is an extremely rare condition, requiring careful differential diagnosis in patients presenting with a papulonodular mass of the eyelid. The clinical relevance lies in its potential to mimic both benign and malignant eyelid tumors, particularly sebaceous gland carcinoma, making accurate histopathological evaluation essential to avoid a misdiagnosis.

## Introduction

Folliculosebaceous cystic hamartoma (FSCH) is a unique cutaneous hamartoma comprising follicular, sebaceous, and mesenchymal elements [1]. A hamartoma is a benign tumor-like malformation composed of disorganized mature tissues normally present at the site of origin. FSCH has been deemed exceedingly rare since the initial report of five cases by Kimura et al. in 1991 [1]. Nevertheless, several clinicians have posited that FSCH may be more prevalent than these statistics suggest, perhaps due to its inadequate recognition, both clinically and with histopathology [2, 3]. Despite a large case series of FSCH being reported [4], there has been only one case report of eyelid FSCH in English literature [5]. To the best of our knowledge, we report the second case of eyelid FSCH in a 66-year-old female patient managed with surgical excision and eyelid reconstruction.

## Case presentation

**Figure 1:**
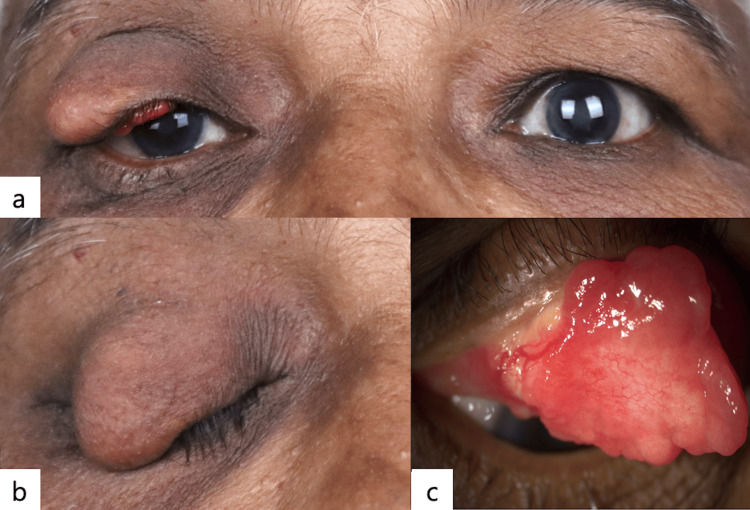
Clinical photograph (a) Preoperative clinical photograph showing a right eye upper eyelid mass with mild ptosis; (b) Closed right upper eyelid showing an overhanging mass with no change in the appearance of the skin; (c) Everted right upper eyelid showing a sessile fungating mass over the tarsal surface with a prominent vessel and yellowish deposits.

**Figure 2:**
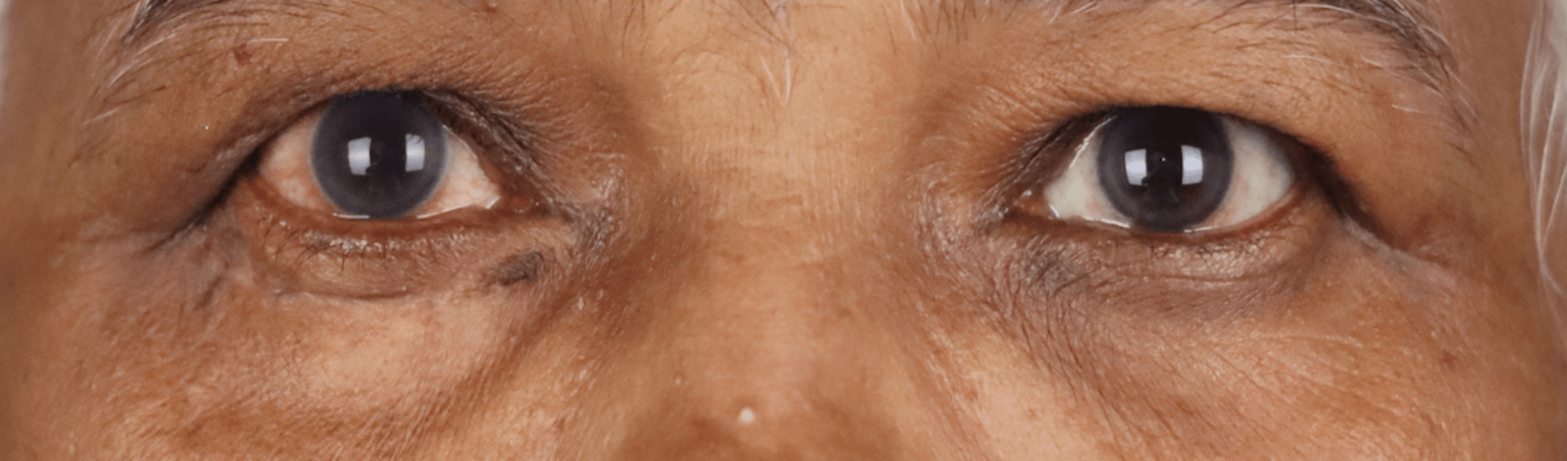
Postoperative clinical photograph at two months following excision and reconstruction showing good symmetry.

**Figure 3:**
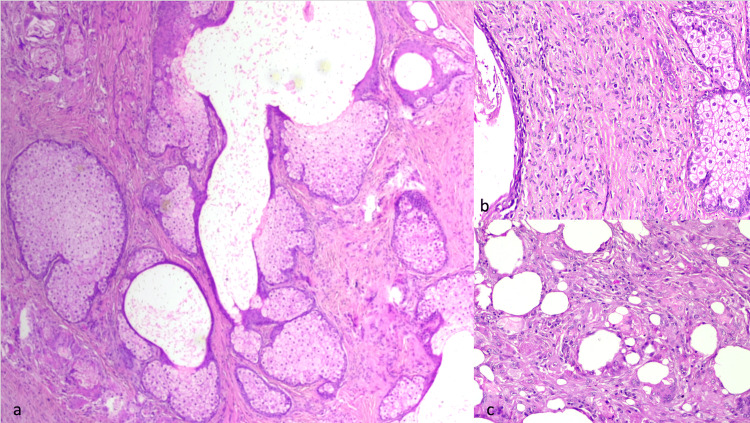
Histopathological microphotograph (a) Photomicrograph showing central dilated cystic space (primary follicle) lined by attenuated squamous epithelium and containing sparsely distributed keratin flakes with conglomerate of numerous sebaceous lobules adjacent to cystic space (H & E stain, 100X); (b) Fibrous, cellular, spindled stroma with sebaceous lobules (H & E stain, 400X); (c) Photomicrograph showing adjacent fat cells (H & E, 400X)

A 66-year-old lady presented with a painless nodule over the right upper eyelid that had been gradually progressing for six months. She had no complaints of irritation or redness in the eye. On examination, her best corrected visual acuity was 20/20; N6 in both eyes. External examination revealed a firm, non-tender, lobulated, well-circumscribed mass measuring 25×15×12 mm (Figure 1a). There was no associated loss of eyelashes. The overlying skin was smooth (Figure 1b). The tarsal surface showed a sessile fungating mass with a prominent vessel and yellowish deposits (Figure 1c). Regional lymph nodes were not enlarged. Ocular examination revealed no abnormalities in the anterior and posterior segments of the left eye, and the posterior segment of the right eye was within normal limits. The differentials considered were a benign sebaceous eyelid tumour or a sebaceous gland carcinoma. Given the lesion’s atypical morphology, such as yellowish deposits, a prominent vessel, but no loss of eyelashes or presence of ulceration, there was a diagnostic uncertainty. Due to this overlap in clinical features and a potential for malignancy, a complete excision of the mass was undertaken for a definitive histopathological diagnosis. The mass was excised in toto with 3mm margins, and the right upper eyelid was reconstructed by a Cutler-Beard procedure (Figure 2). Histopathology showed a well-circumscribed, unencapsulated lesion with a central dilated cystic space lined by attenuated squamous epithelium, containing sparsely distributed keratin flakes, and surrounded by numerous sebaceous lobules (Figure 3a). The background fibrous stroma showed numerous foamy histiocytes, smaller empty dilated cystic spaces, foreign body giant cells, spindle cells, and fat spaces (Figures 3b, 3c). There was no secondary follicle in the wall of the cystic space. These features are compatible with the diagnosis of FSCH. The patient had good eyelid symmetry and no recurrence at the 16-week follow-up.

## Discussion

FSCH is mainly localized at the cephalic extremity, particularly on the face in two-thirds of cases, with a predilection for the nose and perinasal region, as well as the scalp. It typically presents as a solitary, asymptomatic, flesh-colored papule or nodule, either sessile or pedunculated, having a firm to rubbery consistency. This hamartoma occurs rarely on the limbs [2,6,7], the trunk [8], or the genitals [9-11]. Peri-nipple FSCH has been published [12,13], which is related to sebaceous glands in the areola. Congenital FSCH has also been described [14]. The term "cystic" describes a cyst-like expansion of the follicular infundibulum, rather than representing a true cyst. Due to its relatively non-specific clinical presentation, diagnosing FSCH based solely on clinical findings can be challenging. Therefore, histopathological examination is essential for an accurate diagnosis.

FSCH consists of distinct epithelial and mesenchymal components. The epithelial portion is marked by folliculosebaceous proliferation with a cyst-like infundibular dilation, while the mesenchymal component displays variable fibroplasia, adipocyte metaplasia, and vascular and neural proliferation. Recognizing this lesion is crucial to prevent misdiagnosis with other cystic or cyst-like lesions that feature prominent folliculosebaceous elements and diverse mesenchymal features. In our case, multiple sebaceous gland lobules were radially connected to a cystic structure formed by dilated follicles through sebaceous ducts. Fibrous proliferation, adipocytes, and blood vessels were present between the structures, representing both ectodermal and mesenchymal tissues. This appearance and excessive proliferation of these normal, mature cells led to the diagnosis of FSCH. 

It is important to distinguish it from clinically and histologically similar entities. The evaluation revealed no features suggestive of malignancy, such as infiltrative growth, cytologic atypia, nuclear pleomorphism, increased mitotic figures, or necrosis, thereby excluding the diagnosis of sebaceous gland carcinoma. Sebaceous trichofolliculoma closely resembles FSCH due to shared features like follicular dilation and sebaceous elements; however, unlike FSCH, it typically presents as a depressed lesion with visible terminal or vellus hair emerging from the surface. Histologically, sebaceous trichofolliculoma demonstrates rudimentary secondary follicles, unlike FSCH, which lacks secondary follicle formation. Perifollicular fibroma and fibrofolliculoma differ by their absence of sebaceous components, displaying predominantly fibrous stroma surrounding follicular structures without associated sebaceous lobules. In the series by Wu et al., the criteria for diagnosing FSCH included (1) hyperplasia of the follicular epithelium and sebaceous lobules within a dense fibrous stroma, (2) the presence of either a dilated infundibulocystic structure or the presence of adipose tissue, and (3) the absence of secondary follicles, indicating no differentiation of the lower segment or hair shafts [2]. Identifying these nuances is crucial for an accurate diagnosis of papulonodular lesions in the eyelid and face.

## Conclusions

To the best of our knowledge, this is the second case of FSCH of the eyelid reported in the English literature. Complete surgical excision is curative for FSCH. Nevertheless, follow-up at six months postoperatively and then on an annual basis is advisable to monitor long-term outcomes. Although FCSH has a predilection for the face, particularly the nasal region, involvement of the eyelid is rare, requiring careful differential diagnosis in patients presenting with a papulonodular mass of the eyelid.
